# Entropy and Enthalpy Effects on Metal Complex Formation in Non-Aqueous Solvents: The Case of Silver(I) and Monoamines

**DOI:** 10.3390/e24091253

**Published:** 2022-09-06

**Authors:** Andrea Melchior, Martina Sanadar, Rosita Cappai, Marilena Tolazzi

**Affiliations:** 1 Dipartimento Politecnico di Ingegneria e Architettura, Università di Udine, Laboratori di Chimica, via del Cotonificio 108, 33100 Udine, Italy; 2 Dipartimento di Scienze Chimiche, Fisiche, Matematiche e Naturali, Università di Sassari, via Vienna 2, 07100 Sassari, Italy

**Keywords:** silver(I), acetonitrile, monoamines, complexation, solvation, non-aqueous solvents, enthalpy, entropy

## Abstract

Access to the enthalpy and entropy of the formation of metal complexes in solution is essential for understanding the factors determining their thermodynamic stability and speciation. As a case study, in this report we systematically examine the complexation of silver(I) in acetonitrile (AN) with the following monoamines: n-propylamine (*n-pr*), n-butylamine (*n-but*), hexylamine (*hexyl*), diethylamine (*di-et*), dipropylamine (*di-pr*), dibutylamine (*di-but*), triethylamine (*tri-et*) and tripropylamine (*tri-pr*). The study shows that the complex stabilities are quite independent of the length of the substitution chain on the N atom and demonstrates that, in general, the overall enthalpy terms associated with the complex formation are strongly exothermic, whereas the entropy values oppose the complex formations. In addition, we examined the similarity of the formation constants of AgL complexes of the primary monoamines in AN, dimethylsulfoxide (DMSO) and water, which were unexpected on the basis of the difference between the donor properties of solvents.

## 1. Introduction

The thermodynamics of complex formations between metal ions and nitrogen-donor ligands (N-donor) has been widely studied in water [1,2,3,4,5,6] and in non-aqueous or mixed aprotic solvents [7,8,9,10]. The thermodynamic parameters associated with metal–complex formation were determined to evaluate the influence of solvation and of the electronic/structural/steric properties of the ligands on the complex-stabilities and speciation. The understanding of the thermodynamic aspects of complex formation and the role of solvation is necessary for the correct interpretation of chemical phenomena in solutions and to drive the development practical applications. For example, the knowledge of the species present in aqueous solutions is essential to predicting the role of metal ions in biological systems and the environment, to design metal-sequestering drugs [11,12,13,14] or diagnostic agents [15,16,17]. As far as non-aqueous solutions are considered, the definition of metal speciation in such media is of paramount importance in the development of separation techniques, such as liquid/liquid extraction technologies where auxiliary organic ligands are often employed as selective complexing agents [18].

Silver is currently considered as a “low risk supply” material [19], nevertheless, its demand is increasing for several applications, such as nanomaterial synthesis [20], optical [21], catalytic [22], energy storage [23], adsorptive and medical technologies [24,25,26]. The synergic effect of increasing demand and supply shortages [27,28] stimulated the development of technologies aimed also at recovering silver from secondary sources, such as e-waste [29].

Nitrogen-donor ligands have been shown to be good candidates as chelators for cations, as has emerged in a number of studies, carried out by our group on some relevant metals, silver(I) and other transition or *f*-group cations, especially in dimethylsulfoxide (DMSO) [8,10,13,30,31]. In those works, the structure and donor properties of the ligand, metal charge, solvation/desolvation processes of the reagents and the products were analyzed considering the thermodynamic parameters of complex-formation (Δ*G*°, Δ*H*° and Δ*S*°). On the contrary, other solvents [32,33], such as acetonitrile (AN) [10], the thermodynamics of Ag(I)-N-donor ligands has been less investigated.

With the aim of filling this gap and exploring the coordination of N-donors in AN, we report here the results of a systematic study on the coordination of silver(I) by a series of monoamines: n-propylamine (*n-pr*), n-butylamine (*n-but*), hexylamine (*hexyl*), diethylamine (*di-et*), dipropylamine (*di-pr*), dibutylamine (*di-but*), tri-ethylamine (*tri-et*) and tripropylamine (*tri-pr*). The ligands were selected to analyze the influence of enthalpy and entropy terms in determining the stability and nature of the species formed with ligands with the same donor atom and a variable number of alkyl substituents and alkyl chain lengths.

Complex formation constants were obtained from potentiometric titration experiments, while titration calorimetry was employed for the determination of formation enthalpies [10].

Thermodynamic data obtained in this study are compared with those available in water, DMSO, dimethylformamide (DMF) and propylene carbonate (PC), which differ from AN for their dielectric constants (ε) and donor numbers (DN) [34].

## 2. Materials and Methods

### 2.1. Materials

The ligands (Aldrich, Darmstadt, Germany, >98%) were purified by fractional distillation. AN (Fluka, Darmstadt, Germany, >99%) and the background salt (tetraethylammonium perchlorate, NEt_4_ClO_4_), used to maintain the required 0.1 mol dm^−3^ ionic strength, were purified and dried according to the described procedures [35]. Anhydrous silver perchlorate was obtained by drying AgClO_4_·H_2_O (Aldrich, Germany) as reported in ref. [36].

The metal ion and ligand solutions were prepared by dissolving weighted amounts of the reagents in anhydrous AN, with the ionic medium adjusted to 0.1 mol dm^−3^ with NEt_4_ClO_4_. All the solutions were prepared afresh before use in a MB-150 Braun glove box under atmosphere of dry nitrogen. The water content in the solutions, typically 4–8 ppm, was determined by a Metrohm (Metrohm Italiana Srl, Varese, Italy) 684 KF Coulometer.

### 2.2. Potentiometry

A thermostated potentiometric cell, which was used to carry out all measurements, was maintained at 298.15 ± 0.1 K inside the glove box. The cell electromotive force (e.m.f.) was measured by means of an Amel 338 pHmeter equipped with a silver Metrohm 6.1248.010 ion selective electrode, and a Metrohm 6.0718.000 silver electrode, as reference. Experimental runs consisted of collecting equilibrium data-points when solutions of silver perchlorate (1.00 < C°_Ag*_ < 10.00 mmol dm^−3^ in AN) were titrated with solutions of the ligands (40 < C°_L_ < 400 mmol dm^−3^). In the case of *tri-pr*, higher concentrations of metal ion (C°_Ag_ about 20 mmol dm^−3^) were also investigated to maximize the potentiometric response. Titrations were performed with at least three different initial Ag^+^ ion concentrations and some of them were carried out in duplicate to verify the reproducibility of the system. The Nernstian response of the ion-selective electrode was checked in the 10^−6^ < [Ag^+^] < 10^−1^ mol dm^−3^ concentration range. The silver(I) equilibrium concentrations, were used for the determination of the stability constants of the complexes by using the computer program Hyperquad [37].

### 2.3. Calorimetry

A Tronac model 87-558 precision calorimeter was employed to measure the heats of reaction, modified in such a way that the measure-cell could be assembled inside the glove box to avoid moisture contamination. The calorimeter was checked by titration of tris(hydroxymethyl)aminomethane (tham) with a standard solution of HCl in water. The experimental value of their neutralization heat was found to be Δ*H*° = −47.46 ± 0.20 kJ mol^−1^, in good agreement with the accepted value of −47.53 ± 0.13 kJ mol^−1^ [38].

The calorimetric titrations were performed at 298.15 ± 0.1 K by adding known volumes of ligand to silver(I) solutions with concentrations close to those used for potentiometry. In the case of *tri-et* and *tri-pr*, which formed the weakest complexes among those investigated, the concentration range of metal ion was extended to 20 mM to obtain a more relevant heat signal. To reach higher ligand-to-metal ratios, also “reverse” calorimetric titrations, where the metal ion solution was placed in the burette and the ligand solution in the cell, were carried out. Typically, ~100 mmol dm^−3^ solutions of the silver(I) ion were added to 30–65 mmol dm^−3^ solutions of the ligand.

The heats of dilution of the reactants, determined in separate runs, were negligible. The neat reaction heats were used as input data for the minimization program LETACALPD (a home modified version of the Letagrop program [10,39] for the calculation of the standard formation enthalpies). The simultaneous fitting of the stability constants and formation enthalpies of the data of direct and reverse titrations allowed to determine accurate values of the stability constants and enthalpy values, especially for the less-complexing amine *n-pr*. The cEST tool [40] was used for speciation calculations and statistical analysis.

## 3. Results and Discussion

The best fit of the potentiometric and calorimetric data was achieved for the reactions: Ag^+^ + jL ⇌ AgL_j_^+^ (L = *n-pr*, *n-but*, *hexyl*, *di-et*, *di-pr*, *di-but*, *tri-et*, *tri-pr*) when the values of j = 1, 2, 3 for primary and j = 1, 2 for secondary and tertiary amines were considered in the speciation models. The formation constants and the corresponding thermodynamic parameters (Δ*G*_j_°, Δ*H*_j_° and Δ*S*_j_°) are reported in Table 1 and Table 2 together with the data available in other solvents [8,41,42,43].

**Figure 1:**
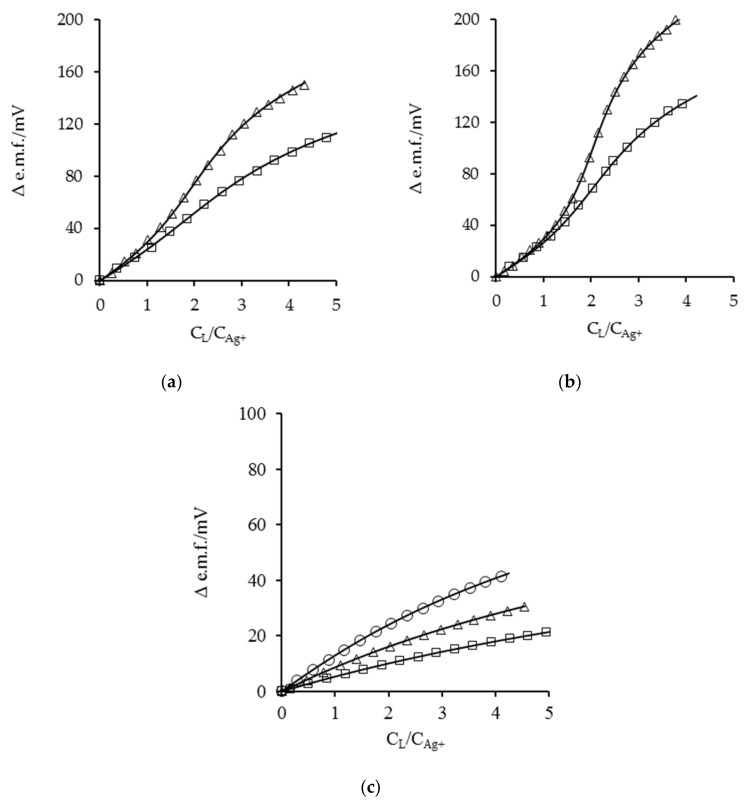
Plot of the observed and calculated Δ e.m.f values obtained for Ag^+^ titrations in AN vs. the total ligand-to-metal (C_L_/C_Ag+_) ratio with (**a**): *n-pr* (*squares*) 2.49, (*triangles*) 9.64 mM; (**b**): *di-pr* (*squares*) 3.28, (*triangles*) 9.81 mM; (**c**): *tri-pr* (*squares*) 4.73, (*triangles*) 9.92, (*circles*) 19.8 mM. Only some of the experimental points, chosen at random, have been plotted.

**Figure 2:**
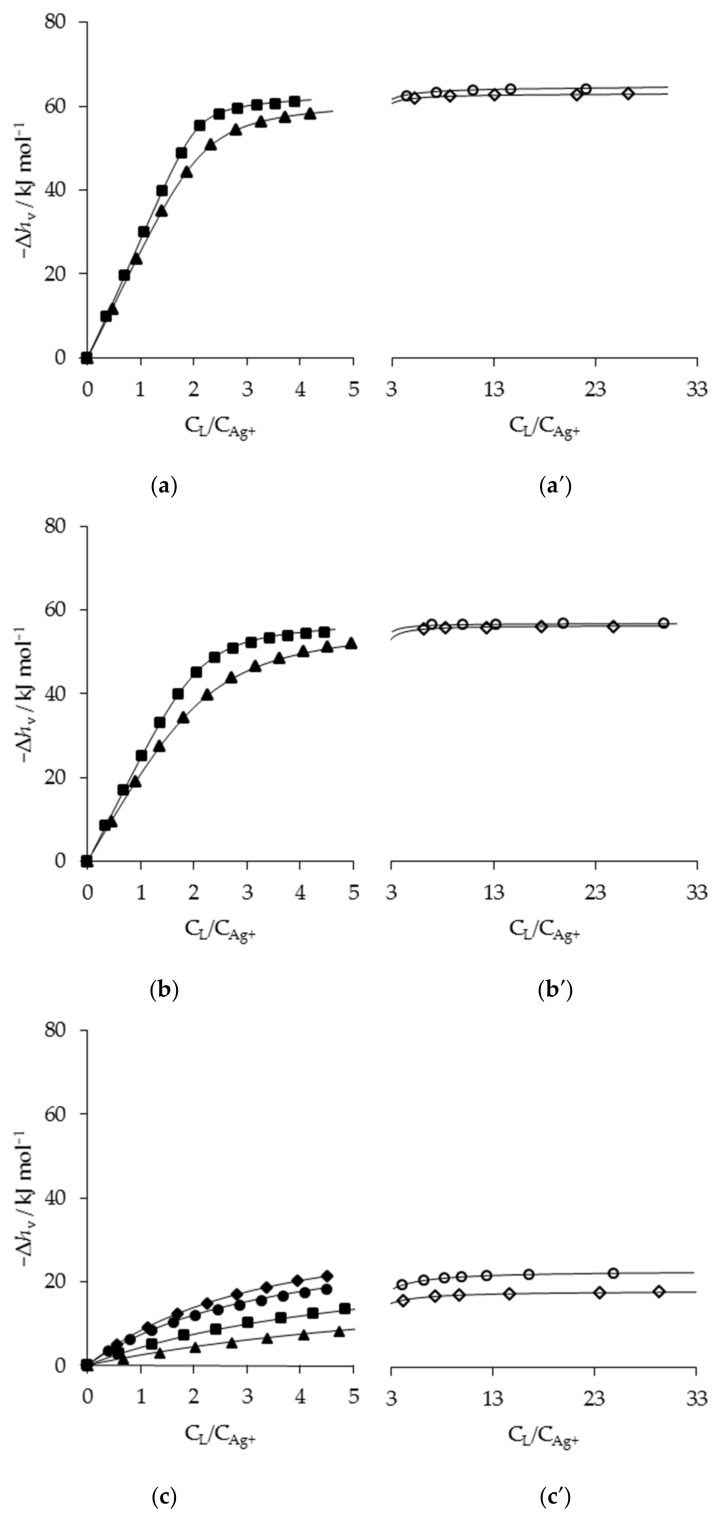
Total enthalpy changes per mole of ligand (direct titrations, x) or added metal (reverse titrations, x′), Δ*h*_v_, as a function of C_L_/C_Ag+_ for Ag^+^ titrations in AN. (**a**): *n-pr* (*triangles*) 2.37, (*squares*) 8.63 mM; (**b**): *di-pr* (*triangles*) 3.14, (*squares*) 9.37 mM; (**c**): *tri-pr* (*triangles*) 2.91, (*squares*) 5.96, (*circles*) 9.86, (*diamonds*) 24 mM. The x′ represent the reverse titrations with ligand in cell (V_cell_ = 20 mL; C°_Ag_ in the burette = 100.4 mM): (**a′**): *n-pr* (*open diamonds*) 35.8, (*open circles*) 60.2 mM; (**b′**): *di-pr* (*open diamonds*) 32.6, (*open circles*) 52.3 mM; (**c′**): *tri-pr* (*open diamonds*) 38.7, (*open circles*) 65.8 mM. Only some of the experimental points, chosen at random, have been plotted.

The AgL and AgL_2_ species are common for all systems, while AgL_3_ is formed only by primary amines. The inclusion of the AgL_3_ species in the models employed in the potentiometric and calorimetric data treatment of primary monoamines always resulted in an improvement of the goodness of fit. It is interesting to note that the literature data [1,8] with primary and secondary-aliphatic monoamines in aqueous and polar non-aqueous solvents are relative only to the AgL and AgL_2_ species. In a potentiometric study by Thaler et al. [44] it was found that *n-but* was able to form only AgL and AgL_2_ complexes in AN (log*β*_1_ = 3.60, log*β*_2_ = 6.95). In our work, the combined potentiometric and calorimetric approach clearly evidenced the formation of AgL_3_. The formation of the AgL_3_ species, emerged by potentiometric data (Figure 1), is also well evident in the silver(I)-*n-pr* system at the higher C_L_/C_Ag+_ ratios reached in the reverse calorimetric titrations: at the end of the calorimetry in Figure 2a′, the percentage formation (relative to total Ag) of AgL_3_ results to be ~10% vs. ~3% in the potentiometric experiments. Interestingly, in the same study [44] the formation constants of the *di-but* complexes in AN (log*β*_1_ = 3.26 and log*β*_2_ = 5.84) are very close to our values (Table 2) within the experimental errors.

The stability of the AgL complexes (Table 1) decreases smoothly on transitioning from primary to tertiary amines (*β_n-pr_* > *β_di-pr_* > *β_tri-pr_*, as an example) and this is evident also from the analysis of Figure 1, where representative potentiometric titration curves for Ag^+^ with *n-pr*, *di-pr*, *tri-pr* are plotted. The inflection near C_L_/C_Ag+_ = 2 (Figure 1a,b) is a consequence of the formation of the complex AgL_2_. The sharpness of the inflection decreases from *n-pr* to *di-pr*, indicating that a less-sTable 1:2 species is formed in the latter case (log*K_2_* = 2.91 for *di-pr vs* log*K_2_* = 3.38 for *n-pr*). In addition, the difference in Δ e.m.f. values at C_L_/C_Ag+_ ≈ 4 (~60–65 mV) is a clear consequence of the formation of the third complex in the case of the less-substituted amine. The curves in Figure 1c result from formation of much less-stable complexes than those of *n-pr* and *di-pr*: in this case the Δ e.m.f. values at C_L_/C_Ag+_ ≈ 4 for the titrations containing about the same concentration of silver(I) (~9.6–9.9 mM) are about 200, 140 and 40 mV for *n-pr*, *di-pr* and *tri-pr*, respectively. Notwithstanding the low variation of potential during titration, the e.m.f. variations are sufficient to also obtain reliable stability constants for *tri-pr*, used as an input in the simultaneous fitting of the stability constants and formation enthalpies of the calorimetric data (see experimental and below). The full lines, calculated with the stability constants listed in Table 1 and Table 2, are in good agreement with the experimental values.

The decrease in complex stabilities on transitioning from primary to more substituted amines is evident not only in AN but also in DMSO (see data for *n-but* and *di-but* in Table 1 and Table 2) and, for alkyl amines different from those here reported, in water [1]. This is in agreement with the common observation that the coordination ability of the nitrogen atoms of alkyl amines is reduced with increasing N-alkyl substitution [8], both in protic and aprotic solvents.

The results of the calorimetric titrations are shown in Figure 2a–c, where the values of Δ*h*_v_ (measured total reaction heat per mole of metal ion) vs. R_c_ = C_L_/C_Ag+_ is plotted for the silver(I)*-n-pr*, *di-pr* and *tri-pr* systems. The experimental data for silver(I)-*n-pr* system in Figure 2a agrees with what expected on the basis of the results of the potentiometric study. The experimental data: (*i*) almost overlap up to R_c_ = 1, according to the nearly quantitative complex formation due to the high value of log*β*_1;_ (*ii*) change their slope up to about R_c_ = 2 (AgL_2_ complex forms in higher quantity when the concentration of AgL in solution is higher); (*iii*) then definitely separate according to the formation of species of very low stability (AgL_3_ in Table 1). Data for the reverse titrations, shown in Figure 2a′, demonstrate that the separation persists even at higher C_L_/C_Ag+_ ratios (circles and diamonds). The minimization of all the experimental data obtained with direct and reverse titrations, allowed us to obtain an estimate of log*β*_3_ and Δ*H°*_3_. In this treatment, the log*β*_j_ (j = 1, 2) obtained in the separated potentiometric data treatment were maintained as fixed input parameters while the complexation enthalpies (Δ*H°*_j_, j = 1–3) and the log*β*_3_ were changed by the minimization program to obtain the best fit of experimental data (full lines in the plots). The results of the calorimetric titrations for *di-pr* system agree with the formation of only two successive complexes, as suggested by the potentiometric data treatment. In this case, another approach similar to the previous one was attempted in order to test the formation of a third complex. However, the data treatment produced completely unreliable results. Hence, the full lines in Figure 2b,b′ were calculated by admitting the presence of only two successive complexes in the solution.

The calorimetric titrations for the tri-substituted amines (*tri-pr*, Figure 2c) are in line with what was obtained in the potentiometric study. Apparently, the calorimetric data are characteristic for the formation of one or two successive complexes. The presence of only one or two complexes in the solution was tested by LETACALPD. The more reliable results were obtained for the formation of two complexes. Hence, the log*β*_j_ and Δ*H°*_j_ (j = 1, 2) listed in Table 2 were obtained with log*β*_1_, obtained from potentiometric data treatment, as fixed parameters, and Δ*H°*_1_, log*β*_2,_ and Δ*H°*_2_ as variables.

The solid lines in Figure 2 represent the values of Δ*h*_v_ calculated with the stability constants and reaction enthalpies listed in Table 1 and Table 2. The good fit between calculated and experimental values confirms the mutual consistency between the potentiometric and calorimetric measurements.

A common feature of complexation reactions in AN, water and other solvents here reported is that complex formation is enthalpy-stabilized in all solvents, the entropy term always being negative (except for the first complexation step for *n-pr* and *n-but* in water). This is characteristic of the formation of metal–ligand covalent bonds and results from the relatively weak solvation of the ligands involved in complex formation and the absence of charge neutralization [8].

As far as Ag(I) solvation is concerned, the general picture arising from experimental and theoretical techniques in the past years is that the ion is tetrahedrally coordinated in DMSO [45,46], DMF [46] and AN [46,47,48]. The coordination of silver ion in water is somewhat less defined, as recent studies proposed in one case a “2 + 2” water coordination [45] and, in another, a linear hydration structure with second shell of water weakly interacting with the metal ion [49]. The available transfer functions of Ag(I) ion from AN to the solvents of concern [50] are listed in Table 3, together with the solvent dielectric constants (ε) and donor numbers (DN) [34]. Data in Table 3 can be useful to discuss the energetics of metal ion desolvation upon complexation.

The Gibbs free energy of transfer in Table 3 shows the following order of affinity of Ag(I) for the different solvents: DMSO > AN > DMF > water > PC. Thus, if only the Ag(I) solvation was considered, a decrease in the stability of metal–amine complexes on transitioning from water to DMF, AN and DMSO and an increase in stability on transitioning from water to PC are expected. In contrast to what was expected on the basis of free energy of transfer of Ag^+^ from AN to water (Δ*G*°_tr(AN__→W)_ = 24.1 kJ mol^−1^, Table 3), the results in Table 1 and Table 2 show that the stabilities of the monoalkylated amines are similar in water and AN and also that they are nearly independent of the length of the substitution chain, both in AN and in water. This is due to the fact that, in addition to the energetic cost related to the desolvation of the metal ion, the thermodynamic parameters associated with the complex formation originate from a sum of other processes: the desolvation of the ligand, the formation of metal–ligand bonds, the solvation of the complex and solvent reorganization [7,8].

**Figure 3:**
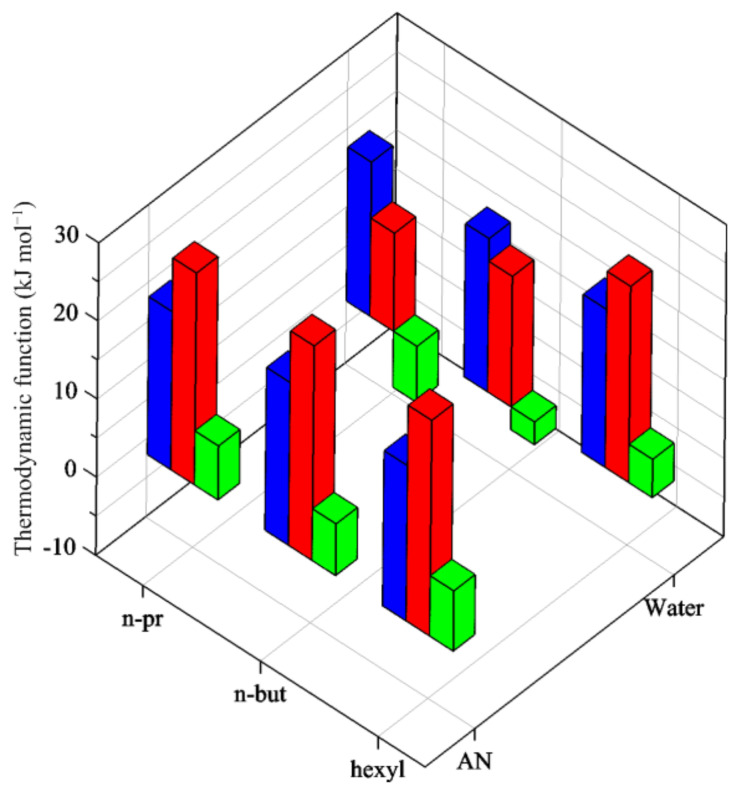
Stepwise thermodynamic functions, −Δ*G*°_K1_ (blue), −Δ*H*°_K1_ (red), −TΔ*S*°_K1_ (green), for the reaction Ag^+^ + L ⇌ AgL (L= *n-pr*, *n-but*, *hexyl*) in AN and water.

The similar stabilities (Figure 3) of the complexes in water and AN (on average −19.8 and −20.3 kJ mol^−1^ in water and AN, respectively) are the consequence of very different enthalpy and entropy contributions. The similar values of Δ*G*°_1_ in AN are the result of the Δ*H*°_1_ and TΔ*S*°_1_, which are almost equal for all amines, as a consequence of similar solvation/desolvation effects of ligands and complexes.

In water, the similar stabilities result from a more and more favorable reaction of enthalpy accompanied by entropic contributions which change from positive for *n-pr* and *n-but* to negative for *hexyl* (Figure 3). It should be noted that the basicity of primary amines has been shown to be little affected by the length of alkyl substituent in water [8,51]. Therefore, the desolvation of the ligand and the solvent reorganization due to the metal complex formation seem to be the major cause of such results. For the less hydrophobic *n-pr*, the effect of the ligand desolvation is more important and the energy cost of this process reflects in a lower (less favorable) reaction enthalpy counterbalanced by a disorder gain. This entropy/enthalpy compensation in water (also with respect to AN) becomes less important as the alkyl chain length increases.

**Figure 4:**
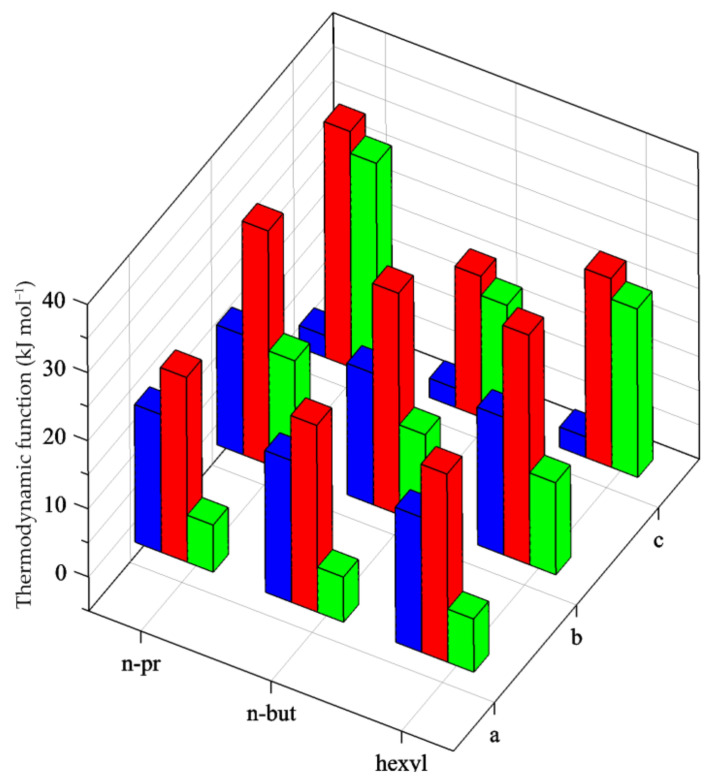
Stepwise thermodynamic functions, −Δ*G*°_Kj_ (blue), −Δ*H*°_Kj_ (red), −TΔ*S*°_Kj_ (green), for the following complex formation equilibria in AN: (a) Ag^+^ + L ⇌ AgL; (b) AgL + L ⇌ AgL_2_; (c) AgL_2_ + L ⇌ AgL_3_ (L = *n-pr*, *n-but*, *hexyl*).

The first stepwise stability constant for the formation of silver(I) with primary monoamines, *K*_1_, is lower than *K*_2_, in AN but also in water and DMSO. The complete set of thermodynamic functions for the reactions concerning the second complexation step reveals that this, apparently anomalous, stability trend results from a balance between the favorable enthalpy of reaction and unfavorable entropy. The stepwise enthalpy (−Δ*H*°_K2_ > −Δ*H*°_K1_) and entropy (−Δ*S*°_K2_ > −Δ*S*°_K1_) trends can be interpreted as reflecting the greater desolvation occurring at the first step of complexation, which makes Δ*H*°_K1_ less exothermic than Δ*H*°_K2_, in spite of the certainly greater metal–ligand interaction in the first complexation step. This is in line with the entropy values which, in the second step, are more than the double of the negative value of the first stepwise value Δ°_K1_ for the monoalkylated amines in Table 1 and in Figure 4, where the stepwise thermodynamic parameters are reported for *n-pr, n-but, hexyl* systems.

These higher stabilities of the AgL_2_ species with respect to AgL (L = primary monoamine or ammonia), and the associated enthalpy and entropy terms were previously [8] explained by assuming that: (*i*) the starting solvated Ag(I) ion was tetrahedrally coordinated in all solvents (*ii*) when the AgL species was formed, a change of the metal coordination geometry occurred, from tetrahedral to linear, causing the release of three solvent molecules with the associated energy cost due to the metal–solvent bond breaking and entropy gain due to the large increase in degrees of freedom in the system (*iii*) when AgL_2_ was formed, only one solvent molecule had to be displaced by the second ligand, which explained the more negative stepwise enthalpy and entropy terms. If the assumption (*i*) remains acceptable for the aprotic solvents [45,46,47], it becomes more nuanced in water, where the metal ion is “quasi-linear” with only two strongly coordinated water molecules [45,49]. Thus, the observed thermodynamic parameters could also be due to the additional effect of the breaking and reorganizing of the hydrogen bond network formed by the second shell water molecules with the hydrated ion when the ligand enters the coordination shell. This could also explain the more favorable entropic terms (positive in the case of Δ*S*°_1_ for *n-pr* and *n-but*) in water.

The trend in enthalpy and entropy may also explain the low stability of the third complex, which is especially destabilized by the entropic term (Table 1 and Figure 4, green bars). The enthalpy associated with the third formation step is high, almost of the same magnitude as in the first two steps, whereas the Δ*S*°_K3_ is much more negative. This agrees with the fact that a strong Ag-N bond is always formed, but unfavorable entropy changes in the coordination sphere of the metal ion, mainly connected with the amine dimensions, occur which almost completely compensate the complexation enthalpy.

AgL_3_ species were not detected in the systems with di- and tri-substituted amines. In this case, the entropic effects following the formation of the 1:3 complexes could be so important as to exceed the corresponding enthalpy gain which, incidentally, becomes less and less favorable with the increase in the number of substituents in amines [8].

Evidently, the third complexation step with these more sterically hindered ligands, entropy effects become even more important and strongly counteract the enthalpy gain, becoming less and less favorable due to decreased N-donor properties with respect to primary ones.

As a matter of fact, the stability constants of the same AgL complexes in AN and DMSO (*n-but* and *di-but*) slightly differ each other as already found for other amines in the two solvents and rather unexpected on the basis of the Ag(I) ion thermodynamic parameters of transfer (Table 3). This was recently discussed [10] in a detailed analysis of the thermodynamic data for the transfer from AN to DMSO of all the compounds participating to the complex formation between Ag(I) and ethylenediamine (*en*) [52]. The result of that study [10], limited to *en*, but reasonably extendable to other amines, was that the comparable stability of Ag-*en* complexes in the two solvents is due to the high value of the transfer enthalpy of the complexes from AN to DMSO, which is only partially compensated by the decrease in order that accompanies their formation in DMSO. Once again, in the present work, the trend in AN and DMSO is confirmed. Finally, the trend of the thermodynamic parameters in DMF (*n-but* in Table 1) and PC (*tri-et* in Table 2), as compared to AN, seems to reflect, in those cases, the prevailing importance of the Ag(I) ion solvation on the complexation thermodynamics.

## 4. Conclusions

The thermodynamics of complex formation between silver(I) ion and a series of neutral monoamines has been systematically studied in AN. In general, the enthalpy terms associated with the complex formation are strongly exothermic, whereas the entropy values oppose the complex formation. Notably, the formation constants for the AgL complexes with primary monoamines in AN, DMSO and water are quite similar. This result is unexpected on the basis of the considerable difference between the metal ion solvation in the above solvents. This study confirms that the complex stabilities are the result of a balancing of different effects which, although different in the various solvents, lead to similar values of log*β*_1_. In particular, the stronger solvation of the ligands in water compensates the lower metal solvation with respect to AN. The enthalpy and entropy values obtained for the formation of silver(I) complexes with primary amines in the aprotic solvent AN also seem to originate from different processes with respect to those presiding the complex formation in water. As the starting geometry of the metal ion is different in the two solvents, the role of breaking the network of hydrogen bonds may be at the origin of the much more favorable entropy (and unfavorable enthalpy) of formation in water with respect to the aprotic solvent. Overall, the net result is a similar stability of the AgL complexes in water and AN (Δlog*β*_1(AN − water)_ < 0.22).

The stability of AgL complexes in DMSO, similar to that in AN, despite the negative Δ*G*_tr(AN →DMSO)_, is due to more negative Δ*H*_K1_ and TΔ*S*_K1_ in DMSO (Table 1 and Table 2). As previously pointed out [10] in a study limited to ethylenediamine complexes, but reasonably extendable to other amines, the comparable stability of AgL complexes in the two solvents is due to the high value of the transfer enthalpy of the complexes from AN to DMSO, only partially compensated by the decrease in order that accompanies their formation in DMSO.

Secondary amines have a similar trend of thermodynamics functions (−Δ*H*_K1_ < −Δ*H*_K2_ and −TΔ*S*_K1_ < −TΔ*S*_K2_) which are less favorable to the complexation and results in a decrease in stability with respect to primary amines of about 0.5 log units.

The complexes with triamines are less stable than those of primary or secondary ones. This can be reasonably interpreted as a steric/structural effect due to the fact that, when the ligand is bound to the metal, the interaction of the complex with the solvent is no longer favored by hydrogen bonding between the coordinated ligand and surrounding water and thus the complex is less stabilized with respect to the same species with primary/secondary amines.

## Figures and Tables

**Table 1:** Overall stability constants and thermodynamic functions (kJ mol^−1^) of silver(I) complexes with primary amines in AN at 298.15 K and I = 0.1 mol dm^−3^; estimated three standard deviations in parentheses. Data at 298.15 K in DMSO [8], DMF [8] and water [41] are inserted. Charges of the species omitted for clarity.

Amine	Solvent	Species	log*β*_j_	−Δ*G*_j_°	−Δ*H*_j_°	−TΔ*S*_j_°
*n-pr*		AgL	3.54 (16)	20.2 (9)	27.1 (4)	6.9
	AN	AgL_2_	6.92 (14)	37.8 (8)	61.4 (4)	23.6
		AgL_3_	7.19 (20)	41.0 (1.1)	96 (6)	55
*n-but*	AN	AgL	3.65 (1) [8]	20.8 (1)	27.4 (1)	6.6
		AgL_2_	7.04 (1) [8]	40.1 (1)	59.9 (1)	19.8
		AgL_3_	7.53 (19) [8]	42.9 (1.1)	80.6 (7)	37.7
*hexyl*		AgL	3.48 (13)	19.8 (7)	27.6 (8)	7.8
	AN	AgL_2_	7.02 (12)	40.0 (7)	61.3 (2)	21.3
		AgL_3_	7.54 (16)	43.0 (4)	89 (2)	46
*n-but* [8]	DMSO	AgL	3.58	20.46	31.4	10.9
		AgL_2_	7.34	41.88	71.5	29.6
*n-but* [8]	DMF	AgL	4.80	27.4	36.3	8.9
		AgL_2_	9.59	54.7	86.5	31.8
*n-pr* [41]	Water	AgL	3.57	19.71	12.55	−7.16
		AgL_2_	7.7	42.47	50.21	7.74
*n-but* [41]	Water	AgL	3.43	19.60	16.70	−2.90
		AgL_2_	7.97	45.50	52.70	7.20
*hexyl* [41]	Water	AgL	3.66	20.21	25.10	4.89
		AgL_2_	7.83	43.18	54.39	11.21

**Table 2:** Overall stability constants and thermodynamic functions (kJ mol^−1^) for the complex formation between silver(I) and secondary and tertiary amines in AN at 298.15 K and I = 0.1 mol dm^−3^; estimated three standard deviations in parentheses. Data at 298.15 K in DMSO [8], PC [42] and water [43] are also inserted. Charges of the species omitted for clarity.

Amine	Solvent	Species	log*β*_j_	−Δ*G*_j_°	−Δ*H*_j_°	−TΔ*S*_j_°
*di-et*	AN	AgL	3.17 (12)	18.0 (6)	26.5 (2)	8.5
		AgL_2_	6.03 (10)	34.1 (3)	58.5 (3)	24.4
*di-pr*	AN	AgL	3.12 (1)	17.8 (1)	26.4 (4)	8.6
		AgL_2_	5.87 (1)	33.5 (1)	57.9 (2)	24.4
*di-but*	AN	AgL	3.14 (2)	17.9 (2)	27.9 (1)	10
		AgL_2_	6.09 (1)	34.7 (1)	55.6 (2)	20.9
*tri-et*	AN	AgL	2.20 (2)	12.5 (2)	23.8 (6)	11.3
		AgL_2_	3.35 (2)	19.3 (3)	49.2 (9)	29.9
*tri-pr*	AN	AgL	1.66 (8)	9.4 (5)	23.2 (8)	13.8
		AgL_2_	2.45 (11)	14.0 (6)	42 (2)	28
*di-but* [8]	DMSO	AgL	2.66	15.17	31.80	16.63
		AgL_2_	5.16	29.43	61.60	32.17
*tri-et* [42]	PC	AgL	7.83	-	-	-
		AgL_2_	10.83	-	-	-
*di-et* [43]	Water	AgL	3.11	-	-	-
		AgL_2_	6.43	-	-	-
*tri-et* [43]	Water	AgL	2.33	-	-	-
		AgL_2_	4.29	-	-	-

In ref. [43] the ionic medium is KNO_3_ 1.0 mol dm^−3^.

**Table 3:** Dielectric constants (ε) and donor numbers (DN) of some organic solvents [34] with the free energies, enthalpies and entropies of transfer of silver ion from AN to the concerned solvent [50] (Δ*G*°_tr(AN→solv)_, Δ*H*°_tr(AN→solv)_ and TΔ*S*°_tr(AN→solv)_ respectively) at 298.15 K. All transfer thermodynamic parameters are in kJ mol^−1^.

	ε	DN	Δ*G*°_tr_	Δ*H*°_tr_	TΔ*S*°_tr_
AN	35.94	14.1	-	-	-
PC	64.92	15.1	42.9	4.2	−38.7
Water	78.36	18.0	24.1	52.7	28.6
DMF	36.71	26.6	6.9	9	2.1
DMSO	46.45	29.8	−7.9	1	8.9

## Data Availability

Not applicable.
